# Comprehensive immune profiling reveals substantial immune system alterations in a subset of patients with amyotrophic lateral sclerosis

**DOI:** 10.1371/journal.pone.0182002

**Published:** 2017-07-25

**Authors:** Michael P. Gustafson, Nathan P. Staff, Svetlana Bornschlegl, Greg W. Butler, Mary L. Maas, Mohamed Kazamel, Adeel Zubair, Dennis A. Gastineau, Anthony J. Windebank, Allan B. Dietz

**Affiliations:** 1 Human Cellular Therapy Laboratory, Division of Transfusion Medicine, Department of Laboratory Medicine and Pathology, Mayo Clinic, Rochester, MN; 2 Department of Neurology, Mayo Clinic, Rochester, Minnesota, United States of America; 3 Department of Neurology, University of Alabama at Birmingham, Birmingham, Alabama, United States of America; 4 Department of Hematology, Mayo Clinic, Rochester, Minnesota, United States of America; 5 Department of Immunology, Mayo Clinic, Rochester, Minnesota, United States of America; "INSERM", FRANCE

## Abstract

Amyotrophic lateral sclerosis (ALS) is a fatal neurodegenerative disease with a median lifespan of 2–3 years after diagnosis. There are few meaningful treatments that alter progression in this disease. Preclinical and clinical studies have demonstrated that neuroinflammation may play a key role in the progression rate of ALS. Despite this, there are no validated biomarkers of neuroinflammation for use in clinical practice or clinical trials. Biomarkers of neuroinflammation could improve patient management, provide new therapeutic targets, and possibly help stratify clinical trial selection and monitoring. However, attempts to identify a singular cause of neuroinflammation have not been successful. Here, we performed multi-parameter flow cytometry to comprehensively assess 116 leukocyte populations and phenotypes from lymphocytes, monocytes, and granulocytes in a cohort of 80 ALS patients. We identified 32 leukocyte phenotypes that were altered in ALS patients compared to age and gender matched healthy volunteers (HV) that included phenotypes of both inflammation and immune suppression. Unsupervised hierarchical clustering and principle component analysis of ALS and HV immunophenotypes revealed two distinct immune profiles of ALS patients. ALS patients were clustered into a profile distinct from HVs primarily due to differences in a multiple T cell phenotypes, CD3^+^CD56^+^ T cells and HLA-DR on monocytes. Patients clustered into an abnormal immune profile were younger, more likely to have a familial form of the disease, and survived longer than those patients who clustered similarly with healthy volunteers (344 weeks versus 184 weeks; p = 0.012). The data set generated from this study establishes an extensive accounting of immunophenotypic changes readily suitable for biomarker validation studies. The extensive immune system changes measured in this study indicate that normal immune homeostatic mechanisms are disrupted in ALS patients, and that multiple immune states likely exist within a population of patients with ALS.

## Introduction

The role of neuroinflammation in amyotrophic lateral sclerosis (ALS) has long been hypothesized. A variety of recent data has emerged to strengthen the validity of this mechanism[1]. Microglia, astrocytes, and inflammatory leukocytes are thought to be key players in this process. These cells have been found to be altered in autopsies of patients with ALS[2–4]. While it is not clear whether neuroinflammation causes ALS, it is theorized that it helps determine the rate of disease progression. For example, in transgenic SOD-1 rodent models, mutant SOD-1 in motor neurons primarily determines disease onset, whereas mutant SOD-1 in astrocytes and microglia primarily determines the rate of progression[2, 5]. In a series of elegant studies, Appel and colleagues demonstrated in transgenic mouse models that while the immune system did not determine the onset of disease, it strongly influenced the rate of clinical progression[6, 7]. While these mouse models highlight the importance of the immune system in the pathogenesis, the knowledge base of the mechanisms of the disease in humans remain severely limited.

Abnormalities in the peripheral immune system have been documented in patients with ALS (reviewed by Malaspina et al[8]). While it appears that multiple cell types are involved in potentiating neuroinflammation, there are also cellular mediators of the immune system that serve to protect against such inflammation[6, 7]. Therefore, disease progression may be dependent on the balance between these two immune functions. This observation may explain why drugs that target neuroinflammation have been disappointing in clinical trials[9–12]. Many treatment trial failures in neurodegenerative diseases are also likely due to the fact that the disease is advanced or the therapies target the wrong aspects of the immune system. A full accounting of the immune status of these patients and the role it plays in the pathology of the disease is still needed.

To better understand the immune status of the patients with ALS, we took a comprehensive, un-biased, approach. The approach is first based on accurate measurement of leukocytes using cell counts per volume rather than relational measurements (i.e. percentage of a parent leukocyte population). We do this by using quantitative multi-color flow cytometry that measures discrete leukocyte populations (defined as a **leukocyte phenotype**)[13, 14]. Measuring each leukocyte phenotype is the basis for the bioinformatics assessment to identify combinations of leukocyte phenotypes that correlate within groups of patients. This objective accounting of a large collection of leukocyte phenotypes helps to identify novel alterations in the immune system by considering an immune system-wide approach to immunity instead of a reductionist approach that focuses on small subsets of cell types. In other words, multiple immune cells and surface markers (leukocyte phenotypes) are measured simultaneously to generate an overall patient **immunophenotype** that can be used to objectively categorize systemic immune alterations in peripheral blood. Patients with similar immunophenotypes can be further grouped into **immune profiles**, which may then be analyzed to understand independent associations for the pathogenesis or prognosis of patients. We have previously performed this methodology on several different cancer patient populations[13]. From these studies, we found that patients with the same cancer type displayed profound immunological differences that ultimately affected survival. Therefore, we extended this analysis to ALS patients and hypothesized that there might be subgroups of ALS patients with distinct immunological changes and that these groups of patients would associate with different clinical parameters.

## Materials and methods

### Patients

This study was reviewed and approved by the Mayo Clinic Institutional Review Board and all patients provided written informed consent to participate. 80 patients with clinically-possible, probable, or definite ALS by El Escorial Criteria[15] were enrolled. No patients were ventilator-dependent or had any significant medical condition, malignancy, active infection, or use of immune modulating or experimental therapy within 6 months of enrollment. Patients were allowed to take riluzole. At enrollment whole blood samples were obtained to perform immunophenotyping analysis. At each clinic visit, the revised Amyotrophic Lateral Sclerosis Functional Rating Scale (ALSFRS-R)[16] was scored, which is a 12-question survey that assesses functional domains and has a maximum score of 48 when there are no measurable impairments. To generate slopes, ALSFRS-R scores were plotted over time (by months) and a linear regression was used to calculate the slope. Familial patients were classified as definite (1^st^ degree relative with ALS with positive genetic testing when available), probable (non 1^st^ degree relative with ALS or one patient who had autopsy findings consistent with a specific familial form), or possible (1^st^ degree relative with a neurodegenerative disorder (Alzheimer, Parkinson, neuropathy, etc.) as these may be associated with some familial forms of ALS). Survival duration, confirmed in 76/80 patients, was defined from onset of symptoms to death or ventilator-dependence.

### Peripheral blood immunophenotyping by flow cytometry

Peripheral blood samples from 50 age and gender matched healthy volunteers and 80 ALS patients were collected in K_2_EDTA tubes (BectonDickinson) at initial and return visits as indicated. Un-manipulated whole blood was stained directly with antibodies. For a subset of initial patients (n = 17) flow cytometry was performed on the 2 laser, 4-color FACSCalibur (BectonDickinson) with antibody panels previously described[13, 17]. For the remaining 63 patients, flow cytometry was performed with expanded flow protocols on the 3 laser, 10-color Gallios Flow Cytometer (Beckman Coulter). All 10-color procedures, antibodies, flow protocols, instrument settings, and gating strategies have been previously described by Gustafson et al[14]. Some patients were additionally stained with a modified T cell signaling protocol[18] to assess CD28 expression on CD3^+^CD56^+^ T cells. Analysis of flow data was performed using Cellquest (BD) or Kaluza (Beckman Coulter) software.

### Statistical analysis

All graphical representations and statistical analyses were performed using Prism software version 7.0 (GraphPad Software). For multiple t-test comparisons, a false discovery rate (FDR) approach was performed via the two stage set-up method of Benjamini, Krieger, and Yekutieli whereby individual p values were determined with fewer assumptions, each row analyzed individually, and no assumption of consistent standard deviation. As this was an initial biomarker discovery data set, the FDR was set at 10% for comparisons of HVs vs. ALS, Hi vs. Lo ALSFRS-R score, Profile 1 vs. 2, and age for HVs and ALS patients. As such, discoveries must have met the criteria that the p value <0.05 and the q value < 0.10. The un-paired two-tailed t-test and the non-parametric Mann-Whitney tests were used to determine the significance of differences between unpaired groups where appropriate and the Spearman test was used for correlative data. The Kruskal-Wallis ANOVA was performed when 3 or more groups were compared at one time. For the multiple comparisons (Dunn’s post-test), the mean rank of each column was compared to the mean rank of every other column. Survival curves were assessed for statistical significance by the Log-rank (Mantel-Cox) test and the hazard ratio by the Mantel-Haenszel test. Hierarchical clustering and principal component analysis of selected leukocyte phenotypes was performed using Partek Genomics Suite 6.6 software (Partek Inc. St. Louis, MO). Hierarchical clustering was used to identify the immune profiles and the PCA plots were used to delineate the leukocyte phenotypes that contributed to each principle component. 53 phenotypes were selected empirically on their suitability for clustering (phenotypes with very low abundance in HVs, incomplete data sets, or those measured on multiple populations were not used). The ratios of each phenotype from each subject were imported into the Partek software for analysis as previously described[13]. To generate the correlations of principal components with original variables, the principle component analysis was performed in Partek using the dispersion matrix/correlation function with the results reported as component loadings. The Fisher’s exact test was used to compare the distribution of groups.

## Results

### ALS patients exhibit a diverse array of immune alterations

**Fig 1:**
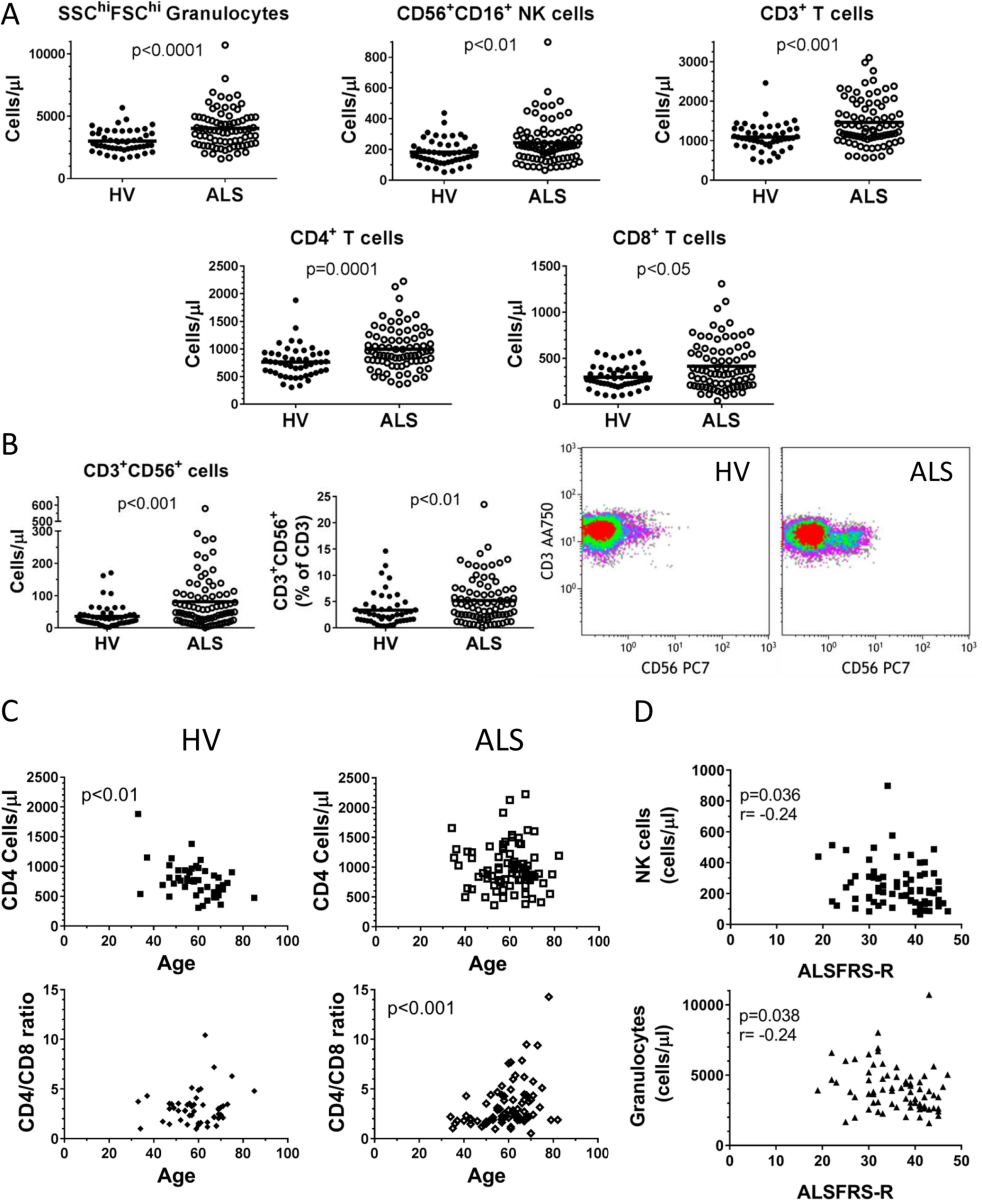
ALS patients exhibit elevated cell counts of granulocytes, NK cells and T cells. Major leukocyte populations from 80 ALS patients and 50 healthy volunteers were assessed by flow cytometry. **A.** Comparisons of granulocytes, NK cells, T cells, CD4^+^ T cells, and CD8^+^ T cells between healthy volunteers (dark circles), and ALS patients (open circles). **B.** CD3^+^CD56^+^ T cells shown as cell counts (cells/μl), percentage of total T cells, and a representative dot plot. **C.** Correlations of CD4^+^ T cells and CD4:CD8 ratios are altered in ALS patients. **D.** NK cells and granulocyte counts inversely correlate with ALSFRS-R score.

We performed multi-parameter flow cytometry on un-manipulated whole blood samples from 50 age and gender matched healthy volunteer controls (HVs) and 80 patients diagnosed with ALS. The patient characteristics are outlined in Table 1. An initial data set was generated from 17 ALS patients from a panel of antibodies designed to quantitate all major leukocyte populations in peripheral blood including granulocytes, monocytes, T cells, B cells, and natural killer (NK) cells[13, 17]. To broaden the number of immune cell phenotypes analyzed, we developed a 10-color, eight protocol panel that measures (at minimum) 116 leukocyte phenotypes in peripheral blood[14]. An additional 63 ALS patients were characterized with this flow panel. A false discovery rate analysis was used to account for the number of false positives when performing multiple comparison testing. We combined the 17 and 63 ALS patient groups and assessed nine leukocyte phenotypes that were measured equivalently as cells/μl and percentages of larger groups, or as ratio: granulocytes (by Side and Forward scatter), CD14^+^ monocytes, CD19^+^ B cells, CD56^+^CD16^+^ NK cells, CD3^+^ T cells, CD4^+^ T cells, CD8^+^ T cells, CD4/CD8 ratio, and CD3^+^CD56^+^ cells. ALS patients exhibited higher peripheral blood cell counts (cells/μl) of granulocytes, NK cells, total T cells, CD4^+^ and CD8^+^ T cells, and CD3^+^CD56^+^ T cells (Fig 1A and 1B), whereas monocytes, B cells, and the CD4/CD8 ratio were not different (S1 Table). CD3^+^CD56^+^ T cells were elevated in ALS patients where both the mean and median cells/μl were more than double than that of healthy volunteer controls (mean: 81.1 cells/μl vs. 36.1 for HVs; median: 53.0 cells/μl vs. 26.4 for HVs). Additionally, CD56^+^ T cells are also elevated as a percentage of total T cells in ALS patients. T cells from ALS patients were 5.2% CD56 positive and HV T cells were 3.4% CD56 positive (Fig 1B middle graph). A representative dot plot of CD56 expression on CD3+ T cells highlights these differences (Fig 1B). These leukocyte phenotypes were also tested to determine whether they correlated with any clinical data of the patients. Unlike in HVs where both CD4 and CD8 counts decrease with age, in ALS patients the CD4 cell counts remained stable with age, which resulted in a significant age-related increase in the CD4/CD8 ratio (Fig 1C). While no leukocyte phenotypes correlated to the slope of progression of the disease, granulocyte and NK cell counts were inversely correlated to the ALSFRS-R score (Fig 1D).

**Table 1:** Clinical characteristics of patients with ALS.

Age (yrs.), n = 80		59.3 (range: 34–82)
Sex		
	Male	45 (56.25%)
	Female	35 (43.75%)
ALS type		
	Sporadic	62 (77.5%)
	Familial	
	Definite	4 (5.0%)
	Probable	3 (3.75%)
	Possible	11 (13.75%)
Site of ALS onset		
	Spinal	54 (67.5%)
	Bulbar	26 (32.5%)
Rilutek use		30 (37.5%)
Time from symptom onset to sample (mos.)		21.6 (range: 3–96)
ALSFRS-R		36.1 (range: 19–47)

Mean values are listed for age, time, and ALSFRS-R score

The use of the eight 10-color flow protocols allowed us to comprehensively examine subpopulations of leukocytes at a much greater detail than with our initial four color panels. From the false-discovery rate analysis of 63 ALS patients and 50 HVs, we found 29 leukocyte phenotypes that were altered in ALS patients (Table 2 (HV vs ALS column) and S1 Table) and met discovery criteria. Cell counts of all four major T cell subsets (CD4^+^, CD8^+^, CD4^+^CD8^+^, CD4^-^CD8^-^), CD3^+^CD56^+^ cells, and γδ T cells were elevated in ALS patients. Furthermore, CD3^+^CD56^+^ cells and γδ T cells were also increased as a percentage of total CD3^+^ T cells. The immunophenotypic changes measured in ALS patients included markers of identity or function (i.e. CD19^+^IgM^+^), differentiation (i.e. stem cell memory CD8^+^ cells) or activation (i.e. CD25^+^CD8^+^ cells).

**Table 2:** List of peripheral blood leukocyte biomarkers relevant to ALS.

Population	Phenotype	HV vs ALS	ALSFRS Hi vs Lo	Correlation to ALS-FRS	ALS Profile 1 vs 2	Correlation to Age	
		Δ*	Δ**	¥	Δ***	HV	ALS
**T cells**	CD3+ T cells (cells/μl)	↑↑↑			↑↑↑	↓↓↓	
** **	CD4+ T cells (cells/μl)	↑↑↑			↑↑↑	↓↓	
** **	CD4+CD25+CD127lo Tregs (cells/μl)			↓	↑↑↑		
** **	CD4+CD25+ (% CD4+)					↓	
** **	CD4+PD-1+ (% CD4+)					↑	
** **	CD4+PD-1+ (cells/μl)				↑↑↑	↓↓	
** **	CD4+CD28+ (cells/μl)				↑↑↑		
** **	CD4+CD28+ (% CD4+)				↓	↓↓	↑
** **	CD4+ Tcm (% CD4+CD45RO+)				↓↓		↑↑
** **	CD4+ Tem (cells/μl)				↑↑↑		↓
** **	CD4+ Tem (% of CD4+CD45RO+)				↑↑		↓↓
** **	CD4+CD45RA+ (cells/μl)				↑↑	↓↓	
** **	CD4+CD45RA+ (% CD4+)					↓	
** **	CD4+CD45RO+ (% CD4+)					↑	
** **	CD4+CD69+ (% of CD4+)						↓
** **	CD4+CD272 MFI						↑
** **	CD8+ T cells (cells/μl)	↑↑			↑↑↑	↓↓	↓
** **	CD8+PD-1+ (% CD8+)					↑	
** **	CD8+PD-1- (cells/μl)				↑↑↑	↓↓	↓
** **	CD8+CD28+ (cells/μl)			↓	↑↑↑	↓↓	↓
** **	CD8+CD45RA+ (cells/μl)				↑↑↑		
** **	CD8+ Tem (cells/μl)				↑↑↑		↓
** **	CD8+ Tcm (cells/μl)				↑↑↑		
** **	CD8+ Tscm (% CD4+CD45RO+)				↑↑↑	↓↓	
** **	CD8+CD25+ (cells/μl)				↑↑		
** **	CD8+CD25+ (% CD8+)	↑↑					
** **	CD8Tscm (% of CD8+CD45RO+)	↓					
** **	CD8T+Temra (%CD8+CD45RA+)				↑↑		
** **	CD8+CTLA4+ (% of CD8+)					↓	
** **	CD8+CD62L+CD27+ (% CD8+CD45RA+)				↓	↓↓	↓
** **	CD4+CD8+ (cells/μl)	↑↑			↑		
** **	CD4-CD8- (cells/μl)	↑↑			↑↑↑	↓↓↓	↓
** **	γδ T cells (cells/μl)	↑↑			↑↑↑	↓↓↓	
** **	γδ T cells (% CD3+)	↑			↑↑↑	↓↓	
** **	CD3+CD56+ (cells/μl)	↑↑↑			↑↑↑		
** **	CD3+CD56+ (% CD3+)	↑			↑↑		
**B cells**	CD19+ B cells (cells/μl)	↑			↑↑↑	↓↓	↓
** **	CD19+CD21- (cells/μl)			↑	↑↑		↓
** **	CD19+CD21- (% CD19+)					↑	
** **	CD19+CD21+ (cells/μl)				↑↑	↓↓	↓
** **	CD19+CD21+ (% CD19+)					↓	
** **	Plasma B cells (% CD19+)	↑↑			↑↑↑		↓
** **	Plasma B cells (cells/μl)					↓↓	↓↓
** **	CD19+CD27+ (% CD19+)				↑↑		↓
** **	CD19+IgM+ (% CD19+)	↓↓↓			↑		↑
** **	CD19+IgD-IgM+ (cells/μl)				↑		↓↓
** **	CD19+IgD+IgM+ (cells/μl)				↑		↓↓
** **	CD19+IgD-IgM- (cells/μl)				↑↑↑		↓↓↓
** **	IgD-IgM- (% CD19+)				↑		↓
** **	Transitional B cells (cells/μl)				↑	↓	
** **	Transitional B cells (% CD19+)	↓					↑
**NK cells**	CD56+CD16+CD3- (cells/μl)	**↑↑**		↓			
**Monocytes**	CD14+HLA-DRlo/neg (cells/μl)						↓
** **	CD14+HLA-DR MFI			↑			↑
** **	CD14+CD86+ (% CD14+)				↓↓		
** **	CD14+CD16- HLA-DR MFI			↑			↑
** **	CD14+CD16- CD80 MFI	↓↓			↓↓		
** **	CD14+CD16- CD86 MFI				↓↓		
** **	CD14+CD16+ HLA-DR MFI	↓					↑
** **	CD14+CD16+ CD86 MFI				↓↓		
** **	CD14+CD16+ CD80 MFI	↓↓			↓↓		
** **	CD14loCD16+ CD86 MFI				↓		
** **	CD14loCD16+ TNFR2 MFI					↑	
** **	CD14loCD16+ CD40 MFI		↑↑	↓			
**Granulocytes**	Granulocytes (SSCby FSC) (cells/μl)	↑↑↑		↓			
** **	CD15+CD16+ (cells/μl)	↑↑↑		↓			
** **	CD15+CD16+ CD66b MFI	↓↓↓			↓↓		
** **	CD15+CD16- (% CD15+)	↓↓					
** **	CD15+CD16-CD49d+CCR3+ CD66b MFI				↓		
** **	CD15+CD16-CD49d+CCR3+ (% CD15+CD16-)	↓↓				↑	
** **	CD15+CD16-CD49d-CCR3- (% CD15+CD16-)	↑				↓	
** **	CD15+ MDSCs (% of PBMCs)				↑		
** **	CD203c+CD63+ (% CD203c+)			↑			
** **	CD203+CD63- (% CD203c+)			↓			
**Other Myeloid cells**	LIN-DR+ DCs (% of PBMCs)	↓↓↓		↑↑	↓↓		
** **	LIN-DR+CD33+CD11c++CD16- (% LIN-DR+)	↑↑			↑↑↑		
** **	LIN-DR+CD33+CD11c++CD16- (cells/μl)				↑↑↑		
** **	LIN-DR+CD33+CD11c++CD16+ (% LIN-DR+)	↓↓			↓↓↓		
** **	LIN-DR+CD33+CD11c++CD16+ (cells/μl)				↓↓↓		
** **	LIN-DR+CD11c dim				↑		
** **	LIN-CD33+HLA-DR- Immature MDSCs (% PBMCs)	↓↓					
** **	LIN-CD33+HLA-DR- Immature MDSCs (cells/μl)				↑↑		
**Lineage Negative cells**	LIN- cells (cells/μl)				↑↑↑		

Selected immunophenotypes are shown. Key: Δ* represents change in value in ALS; Δ** represents change in value in patients with ALSFRS-R Lo (≤32); ¥ ↑ equals positive correlation; ↓ equals inverse correlation; Δ*** represents change in value in patients in Profile 2 compared to Profile 1; ↑ or ↓ equals p value 0.01 to <0.05; **↑↑** or **↓↓** p value 0.001 to <0.01; **↑↑↑** or **↓↓↓** p value <0.001; MFI is the mean fluorescence intensity (geometric mean).

We also tested each of the 116 leukocyte phenotypes to determine whether any other phenotypes associated with clinical parameters. When patients were sub-grouped by ALSFRS-R score, we found that the expression of CD40 on non-classical monocytes (CD14^dim^CD16^+^) was higher in patients with a low ALSFRS-R score (≤ 32) than in patients with a high ALSFRS-R score (>32). Ten leukocyte phenotypes were found to be correlated with ALSFRS-R score in addition to the previously shown NK cells and granulocytes (Fig 1D). Lineage^-^HLA-DR^+^ circulating dendritic cells (Lin^-^DR^+^ DCs as a percent of PBMCs), classical CD14^+^CD16^-^ HLA-DR expression, CD19^+^CD21^-^ immature B cells, and activated basophils (CD203c^+^CD63^+^) declined as the ALSFRS-R score declined, whereas CD4^+^CD25^+^CD127^lo^ regulatory T cells/μl, CD8^+^CD28^+^ cells/μl, CD15^+^CD16^+^ neutrophils cells/μl, non-classical CD14^dim^CD16^+^ CD40 MFI, and basophils (CD203c^+^CD63^-^) increased as the ALSFRS-R score declined (Table 2).

### Two distinct ALS immune profiles reflect different clinical associations

**Fig 2:**
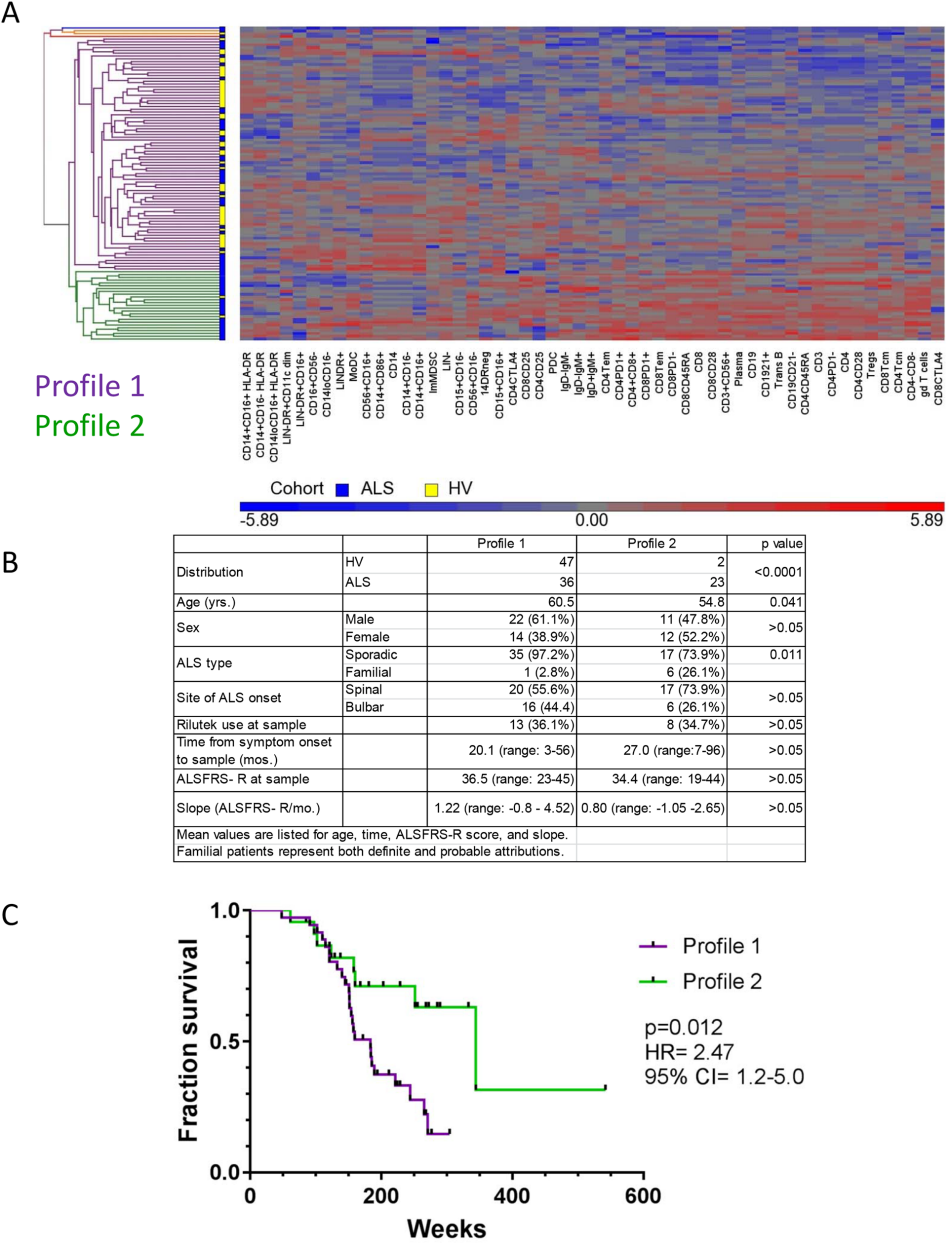
Hierarchical clustering of immunophenotypes reveals subgroups of ALS patients with distinct clinical characteristics. Immunophenotypes of ALS patients and HVs were analyzed by unsupervised hierarchical clustering and principal component analysis as outlined in the methods. **A.** Dendrogram of hierarchical clustering of ALS patients (blue) and HVs (yellow) were grouped into Profile 1 (purple lines) and Profile 2 (green lines). 1 HV and 3 ALS patients were not clustered into a defined profile and 1 ALS patient was excluded due to insufficient data. **B.** Table of clinical characteristics of ALS patients subgrouped by profile. Familial ALS patients included only definite or probable for this analysis. **C.** Survival curves of patients in the two different profiles (Profile 1 with purple line and Profile 2 in green line). HR = hazard ratio and the 95% CI represents the confidence interval of the ratio.

Although a wide variety of immune alterations in ALS patients were identified, relatively few phenotypes associated with clinical parameters. Drawing on our experience with cancer patients where we found unique profiles within patients with the same cancer type, we applied our novel systems-based approach to measuring immune cell populations in ALS patients. From the 63 ALS patients, selected leukocyte phenotypes were incorporated as cells/μl or mean fluorescence intensity. Ratios of immunophenotypes for each subject (both ALS patients and HVs) were calculated by dividing individual leukocyte phenotype by the mean of the 50 HV cohort. Unsupervised hierarchical clustering and principal component analyses were performed on the ratios to generate groups of similar subjects. For this study, the leukocyte parameters/phenotypes/markers include major cell populations (i.e. CD14^+^ monocytes), subsets, parent, or grandparent populations (i.e. CD14^+^CD16^-^ classical monocytes), and other defining cell surface markers that reflect identity and/or function (HLA-DR expression on CD14^+^ monocytes). These parameters can be measured in several ways including cells per volume (cells/μl), as a percent of a parent population, as well as surface intensity on a group of similar cells (mean fluorescence intensity). An immunophenotype encompasses the enumeration of the circulating immune system. As such, the immunophenotype is a snapshot of the immune status of the individual at the time of the blood draw. In this study, the individual’s immunophenotype is comprised of at least 116 measured parameters or characteristics of peripheral blood leukocytes. An immune profile represents a group of individuals who have similar immunophenotypes as determined by the unsupervised hierarchical clustering analysis. The immune profiles reflect the degree and magnitude of immune system alterations relative to a healthy volunteer cohort. An immune profile was determined by groups that contained 5 or more subjects. Fig 2A shows the dendrogram of the distribution of the ALS patients and HVs based on 53 leukocyte phenotypes. The clustering identified two major subgroups in which we defined as Profile 1 and Profile 2. Profile 1 contained 47 HVs and 36 ALS patients whereas Profile 2 contained only 2 HVs and 23 ALS patients (p<0.0001 Fisher’s exact test). Patients in Profile 2 were younger and more likely to have familial ALS (Fig 2B). The patients between these groups did not differ in gender, site of onset, riluzole use, ALSFRS-R score, or slope of progression. However, the patients in Profile 2 were much more likely to survive longer than those patients in Profile 1 (Fig 2C). Patients in Profile 2 had a median survival of 344 weeks compared to 184 weeks for patients in Profile 1 (p = 0.012).

**Fig 3:**
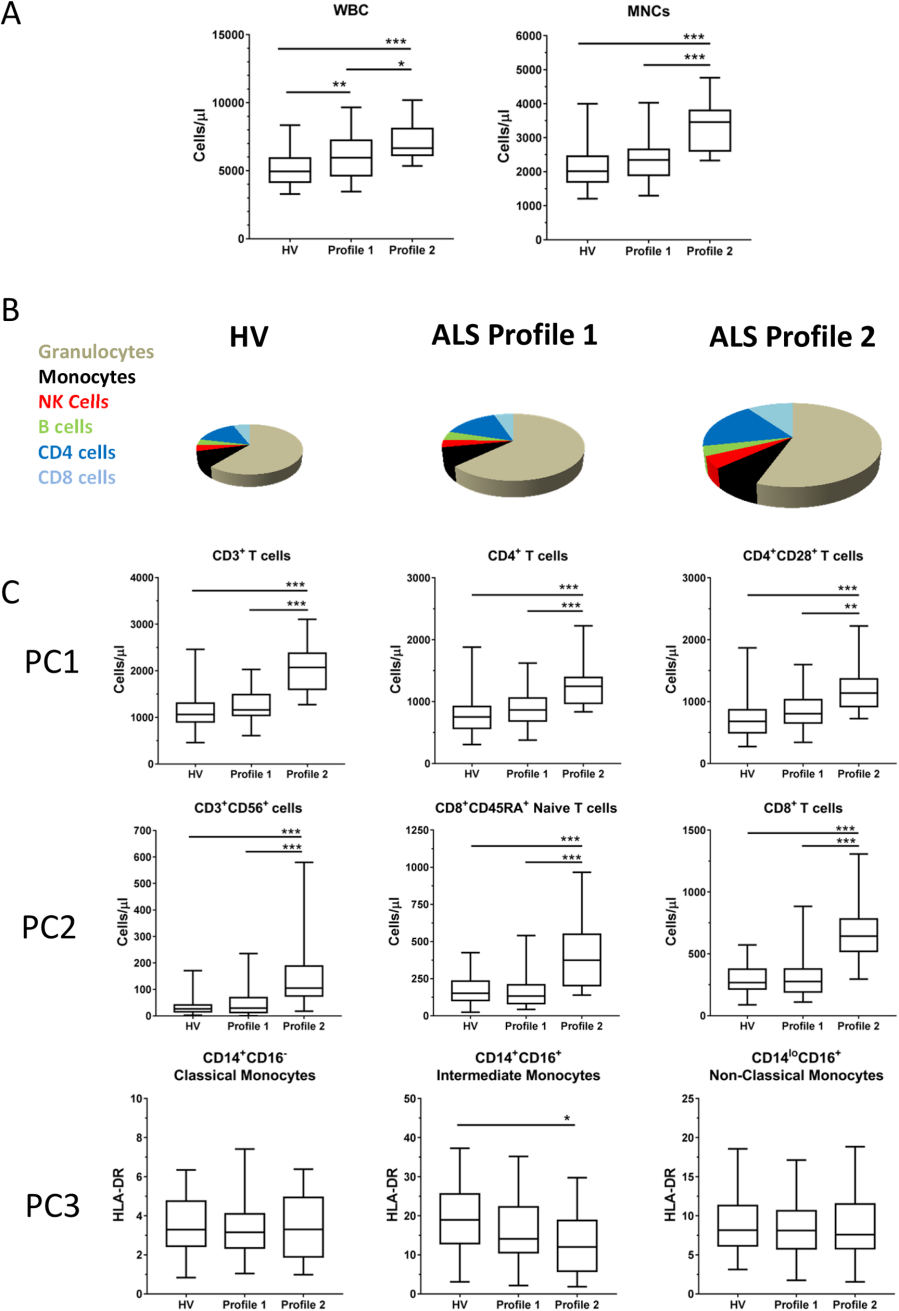
Two immune profiles of ALS patients reveal significant phenotypic differences. Leukocyte phenotypes from HVs and ALS patients sub-grouped into Profiles 1 and 2 were compared. **A.** Box-and-whisker plots of total white blood cells (WBCs) and mononuclear cells (MNCs) are shown. The boxes indicate the 25-75^th^ percentile, the horizontal line indicates the median, and the whiskers represent minimum and maximum values. **B.** Pie graphs depicting the peripheral blood leukocyte compartment of the three groups. The HV pie graph was set to 100% and the other pie graphs were sized in relation to the HV. **C.** Box-and-whisker plots of immunophenotypes associated with principal components. *** = p value <0.001, and * = p value <0.05.

An immune profile identifies groups of patients whose overall immune system is similar. We took these profiles and selected independent leukocyte phenotypes to examine differences between these objectively identified groups. Patients in Profile 2 had both higher total leukocyte counts (7293 cells/μl) and mononuclear cells counts (3357 cells/μl) than both patients in Profile 1 (6070 and 2342 cells/μl respectively) and HVs (5080 and 2073 cells/μl respectively) (Fig 3A). This data is represented pictorially where peripheral blood leukocytes from HVs are represented by a pie graph and the overall size and distribution of phenotypes in the ALS patients are sized in relation to the HV group (Fig 3B). Principal component analysis allowed us to determine which phenotypes were most associated with the variation in the principal components. S1 Fig shows the distribution of ALS patients and healthy volunteers as shown through three major principal components. Principal component (PC) 1 is associated with predominantly T cell phenotypes, PC2 with predominantly CD8 phenotypes, and PC3 mainly myeloid phenotypes (S1 Fig). The top three leukocyte phenotypes from each principal component (S1 Fig) were also compared between patients in Profile 1, Profile 2, and HVs. For PC1 and PC2, the leukocyte phenotypes were consistently elevated in Profile 2 versus the other two groups (Fig 3C). Profile 2 patients, therefore, can be characterized as having strongly elevated levels of CD3^+^ T cells, CD4^+^ T cells, CD8^+^ T cells, CD4^+^CD28^+^ T cells, CD3^+^CD56^+^ T cells, and CD8^+^CD45RA^+^ Naïve T cells. For PC3, HLA-DR expression was measured by mean fluorescence intensity (MFI) on the three types of circulating monocytes[19] however there were only different (lower) on intermediate CD14^+^CD16^+^ monocytes in Profile 2 versus HVs. All remaining immunophenotypes were compared from the 2 profiles and 49 additional leukocyte phenotypes were significantly different between ALS patients in Profile 1 versus Profile 2 (Table 2 (ALS Profile 1 versus 2 column)). Taken together, there are a minimum of two subgroups of ALS patients with clearly identifiable immunological differences associated with distinct clinical characteristics.

### Identification of peripheral blood biomarkers in ALS patients for diagnosis and disease progression

**Fig 4:**
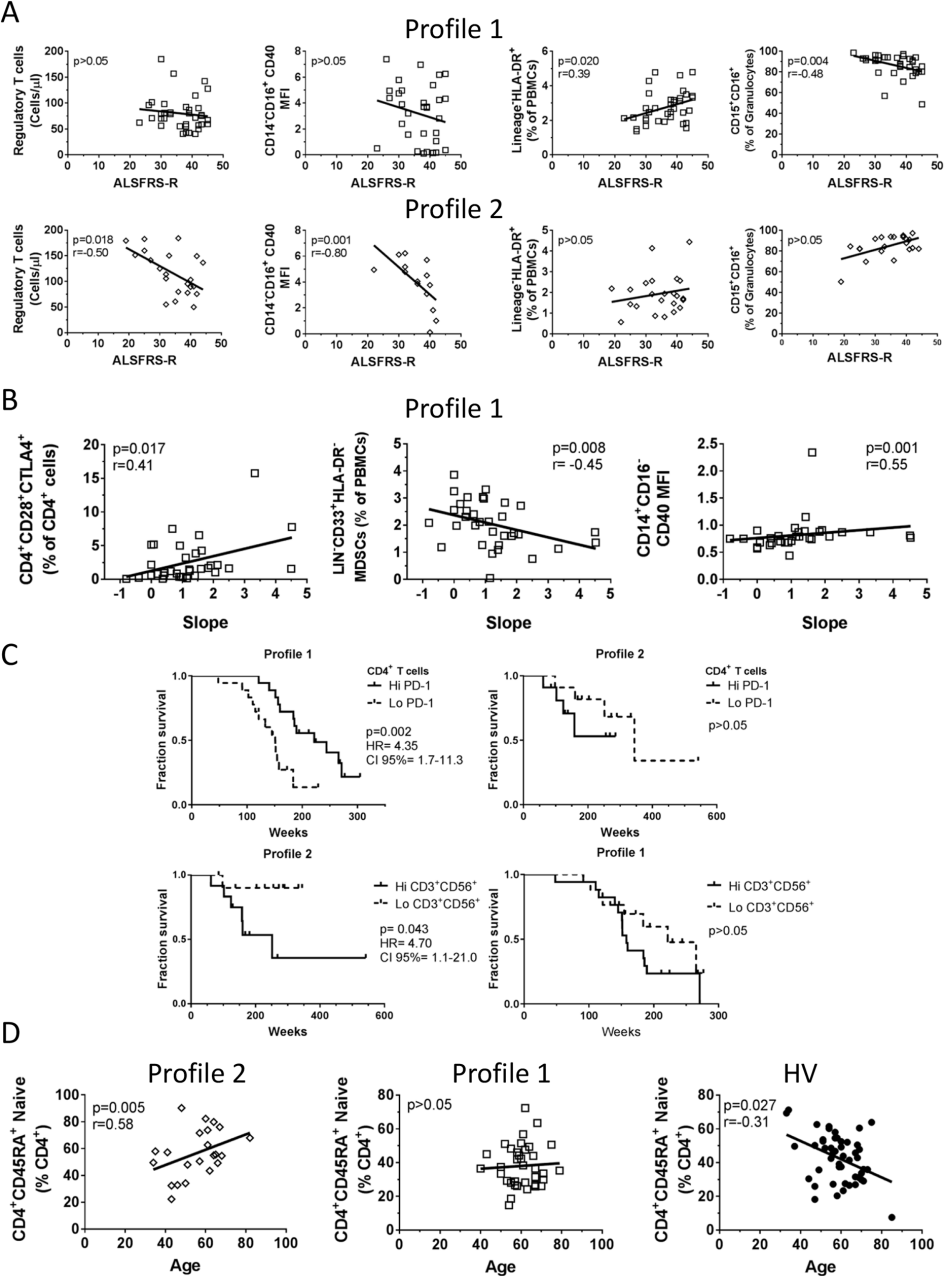
Different immunophenotypic biomarkers associate with clinical parameters in the two ALS immune profiles. Immune phenotypes were plotted against ALSFRS-R score or slope (ALSFRS-R points/month). XY graphs of correlations show p-value and Spearman r value. Lines represent the best fit resulting from linear regression analysis. Closed circles represent healthy volunteers, open squares represent ALS patients in Profile 1, and open diamonds represent ALS patients in Profile 2. **A.** Correlations of selected immunophenotypes to ALSFRS-R score in Profile 1 patients (top row) versus Profile 2 (bottom row). **B.** Correlations of selected immunophenotypes to slope in ALS Profile 1 patients. **C.** Survival curves of patients in each profile sub-grouped by cut-off values for PD-1^+^ CD4^+^ T cells. 19.7% was the cut-off value representing the median value for Profile 1 patients (Hi PD-1≥ 19.7; Lo PD-1< 19.7) and 19.5% was the cut-off value for Profile 2 patients. For CD3^+^CD56^+^ T cells, 104.62 cells/μl was used as a cut-off value for Profile 2 patients and 28.21 cells/μl was used for Profile 1. **D.** CD4^+^CD45RA^+^ naïve T cells show dissimilar age related associations between Profile 2 ALS patients, Profile 1 ALS patients, and healthy volunteers.

Since the two immune profiles were identified in the ALS cohort had a significant difference in survival, we hypothesized that there may be differences in the specific leukocyte phenotypes between the two profiles that may also associate with clinical parameters. CD4^+^CD25^+^CD127^lo^ regulatory T cells/μl and non-classical CD14^dim^CD16^+^ CD40 MFI were identified as phenotypes that demonstrated a strong inverse relationship to the ALSFRS-R score in patients within the Profile 2 group, whereas Lin^-^DR^+^ DCs and CD15^+^CD16^+^ neutrophils (% of total granulocytes) associated with the ALSFRS-R score exclusively for patients in Profile 1 (Fig 4A). The ALSFRS-R score was measured in most patients over time to generate a slope (ALSFRS-R points/month) representing how quickly the disease progressed in each patient. For patients in Profile 1, CD4^+^CD28^+^CTLA4^+^ T cells, and CD40 MFI on classical monocytes positively correlated with the slope, whereas Lin^-^CD33^+^HLA-DR^-^ myeloid derived suppressor cells decreased as the slope increased (Fig 4B). Since we found survival differences between the profiles, we tested whether any phenotypes associated with patient outcomes. A cut-off value was used for each phenotype to then categorize patient survival in those patients that either fell below or above the median value of an immune marker. In patients in Profile 1, the median value for PD-1^+^ CD4 T cells was 19.7%. The survival data of patients whose PD-1^+^ levels were higher than the median were compared against those patients whose PD-1^+^ levels were lower. Patients with a higher PD-1^+^ CD4 T cell percentage survived longer than those that had low levels PD-1^+^ CD4 T cells (222 weeks vs 151 respectively; p = 0.002). This association did not hold true for patients in Profile 2 even though the PD-1 levels were nearly identical (median 19.5%) (Fig 4C, upper panels). For Profile 2 patients, the abundance of CD3^+^CD56^+^ T cells influenced outcome as patients with lower than 104.62 cells/μl survived longer than those that had CD3^+^CD56^+^ T cells higher than 104.62 cells/μl (median survival not yet achieved for patients with low cell counts vs 251 weeks for those with high counts; p = 0.043). CD3^+^CD56^+^ T cell abundance did not appear to influence survival in Profile 1 patients (Fig 4C, lower panels). Although the median was lower than patients in Profile 2 (28.21 cells/μl), we could not identify any cut-off value to yield a significant association in Profile 1 patients. The data taken together indicate that distinct leukocyte phenotypes may influence clinical parameters differently within the two major immune profiles of patients with ALS.

In order to distinguish phenotypic changes affected by age rather than disease, we tested whether the immunophenotypes that age in healthy volunteers similarly change to those with ALS. In healthy volunteers, we found 28 leukocyte phenotypes that were associated with aging (Table 2, last two columns). 21 phenotypes decreased as the age increased whereas six phenotypes increased as age increased. Only eight phenotypes behaved similarly in healthy volunteers and ALS patients (and one phenotype acted differently between the groups), suggesting that 19 phenotypes are not influenced by aging in ALS patients. Additionally, 20 other unique leukocyte phenotypes are influenced by aging in ALS patients that are not regulated similarly in healthy volunteers. One striking example of this is in the accumulation of CD4^+^CD45RA^+^ naïve T cells in ALS patients. ALS patients in Profile 2 show a dramatic increase in the number of CD4^+^CD45RA^+^ naïve T cells (% of total CD4^+^ T cells) in older patients, whereas CD4^+^CD45RA^+^ naïve T cells do not associate with aging in Profile 1 patients (Fig 4D). In contrast, CD4^+^CD45RA^+^ naïve T cells decline as healthy subjects age. Taken together, the data collected in this study identified numerous potential biomarkers that were associated with disease severity, progression of the disease, survival, and age-related leukocyte phenotypes.

**Fig 5:**
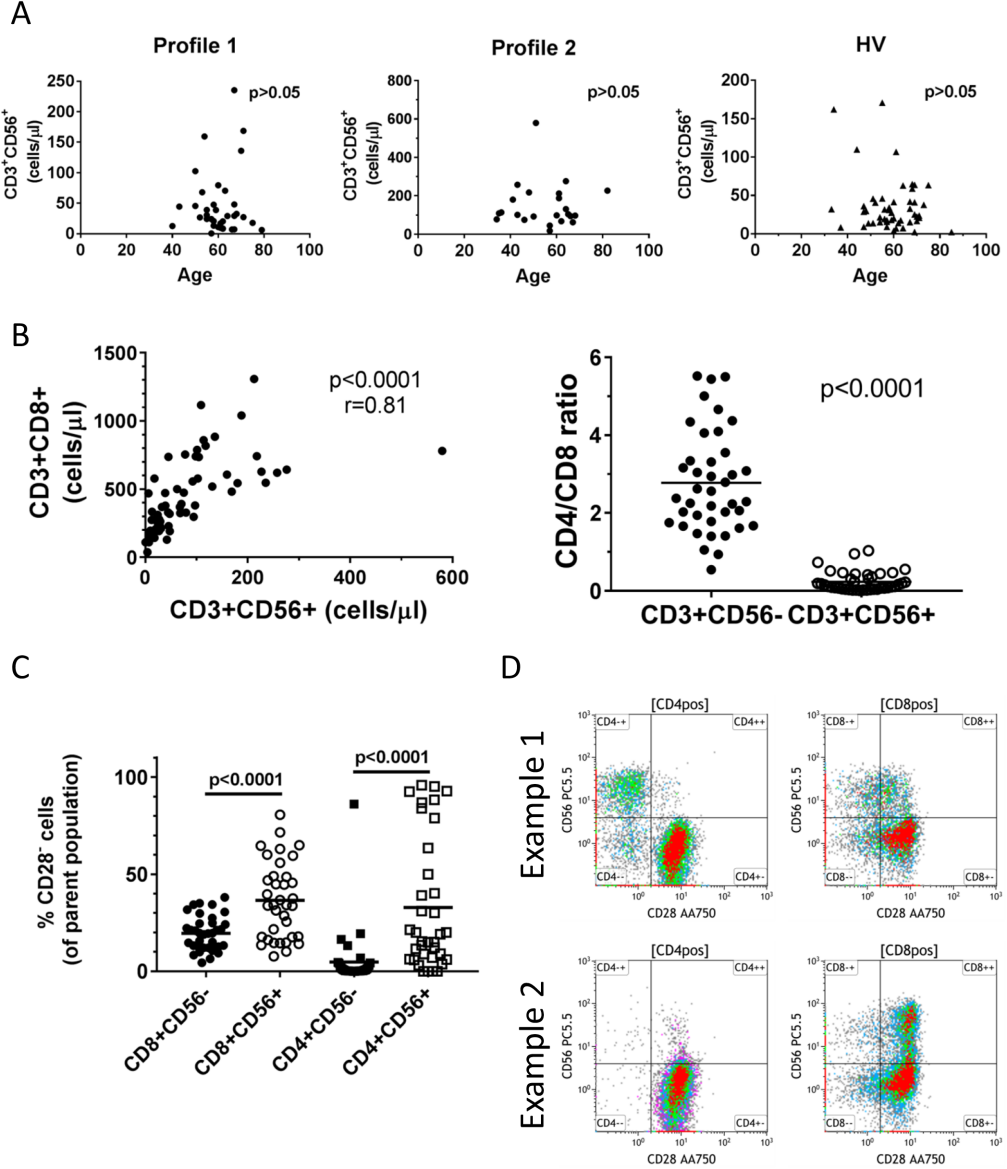
Additional phenotypic characterizations of CD3^+^CD56^+^ T cells. **A.** CD3^+^CD56^+^ T cell counts plotted against age (years) for sub-grouped ALS patients and healthy volunteers. **B.** Correlation between CD8^+^ T cell counts and CD3^+^CD56^+^ T cell counts (left) and the CD4:CD8 ratios are lower in CD56^+^ than CD56^-^ T cells. **C.** Comparison of the percentage of CD28 negative cells in CD56^-^ (filled shapes) and CD56^+^ cells (open shapes) for both CD8 (circles) and CD4 (squares) subsets. **D.** Examples of CD28/CD56 dot plots gated from CD4 and CD8 subsets from two ALS patients.

We performed an extended analysis of the phenotypes with the highest correlation to the principal components, CD3^+^CD56^+^ T cells and HLA-DR on CD14^+^ monocytes, to determine whether they associated with other immune cell phenotypes or clinical characteristics. For CD3^+^CD56^+^ T cells, we have shown that they are elevated in the total ALS cohort with even higher levels in Profile 2 patients. CD3^+^CD56^+^ T cells did not associate with ALSFRS-R score or slope, but they were associated with poorer survival in Profile 2 patients. These cells have previously been associated with chronological aging and gain of CD56 expression while losing CD28 expression presumably through repeated stimulation[20]. We did not find a correlation of the abundance of these cells with age in either ALS patients or healthy volunteers (Fig 5A). CD3^+^CD56^+^ T cell counts from the entire cohort demonstrated the highest correlation with CD8^+^ T cells (Fig 5B). Most CD3^+^CD56^+^ were CD8^+^ as the CD4:CD8 ratio was 0.23 for CD3^+^CD56^+^ cells and 2.78 for CD3^+^CD56^-^ cells (Fig 5B). CD56^+^ T cells from ALS patients exhibited much more CD28 negative cells in both CD4 and CD8 compartments than CD56 negative cells (Fig 5C). However, CD56^+^CD28^-^ cells varied greatly (examples of two ALS patients are shown in Fig 5D) and were not associated with any clinical parameters.

**Fig 6:**
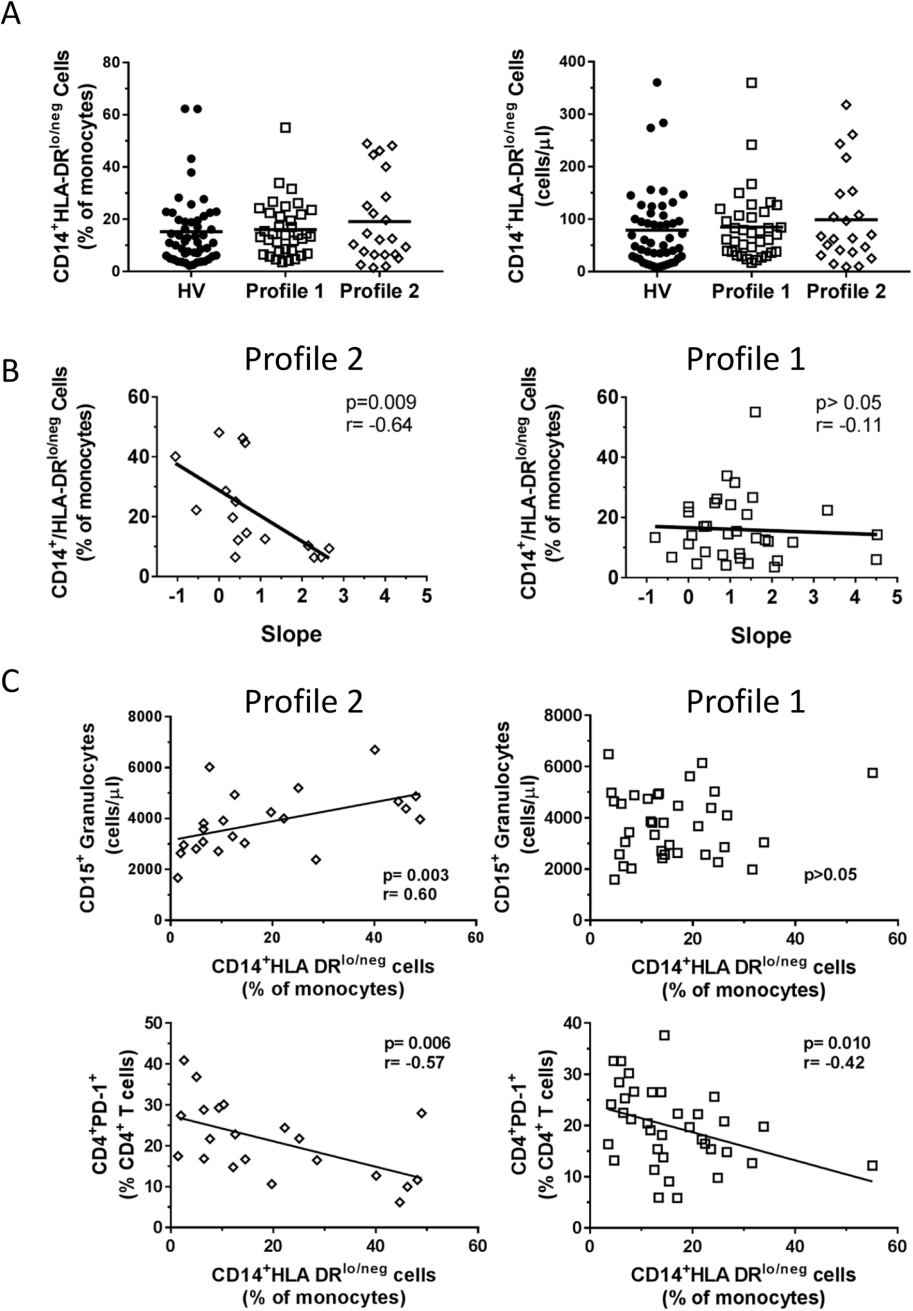
CD14^+^HLA-DR^lo/neg^ monocytes in ALS patients. **A.** Comparison of the percentages and cell counts of CD14^+^HLA-DR^lo/neg^ monocytes in sub-grouped ALS patients and healthy volunteers. **B.** CD14^+^HLA-DR^lo/neg^ monocytes associate with slope in Profile 2 patients but not Profile 1 patients. **C.** Correlations of CD14^+^HLA-DR^lo/neg^ monocytes with other phenotypes in sub-grouped ALS patients (Profile 1: squares; Profile 2: diamonds). XY graphs of correlations show p-value and Spearman r value.

HLA-DR expression on intermediate CD14^+^CD16^+^ monocytes was significantly lower in patients in Profile 2. Intermediate monocytes, along with classical and non-classical monocytes, that express low levels of HLA-DR make up a larger group of cells referred to as CD14^+^HLA-DR^lo/neg^ monocytes. We have previously shown that the presence of CD14^+^HLA-DR^lo/neg^ monocytes, where the number of monocytes with low or no expression of HLA-DR was measured as a percentage and as cell counts, is a very good indicator of immune suppression in cancer patients[21]. HLA-DR expression was measured by flow cytometry in a variety of ways including the mean fluorescence intensity (MFI), as a percentage of monocytes, and as cell counts. In most cases, each parameter is suitable for comparative analyses, but in some conditions, the HLA-DR MFI may be a more sensitive measurement. The gating strategies for these phenotypes have been previously outlined by our group[14]. In contrast to CD3^+^CD56^+^ T cells, we did not detect differences between ALS patients and HVs in HLA-DR expression on monocytes, percentage of HLA-DR^lo/neg^ monocytes, or cell counts (Fig 6A and S1 Table). However, these cells inversely correlated with the ALSFRS-R score in the total cohort of ALS patients (Table 2) and the slope of disease progression in Profile 2 patients (Fig 6B). In Profile 2 patients, CD14^+^HLA-DR^lo/neg^ cells were correlated with the granulocyte cell counts and PD-1^+^CD4^+^ T cells whereas these cells only associated with PD-1^+^CD4^+^ T cells in Profile 1 patients (Fig 6C). CD3^+^CD56^+^ T cell and CD14^+^ HLA-DR phenotypes demonstrate differential abundance and contributions to clinical parameters in ALS patients and thus may reveal mechanistic insights into the pathology of the disease.

## Discussion

The goals of this study were to identify biomarkers that allow improved staging or prognosis of the disease through the discovery of previously unrecognized immune abnormalities in ALS. To that end, we performed multi-parameter flow cytometry on fresh, un-manipulated whole blood to measure 116 leukocyte phenotypes. The broad range of ALS severity was included in order to better determine immunophenotyping changes across the spectrum of disease, including very early disease. Through an unbiased hierarchical clustering approach, we identified (at minimum) two immune profiles in ALS patients with very distinct peripheral blood compositional differences, which corresponded to divergent clinical correlates. Patients that were clustered into Profile 1 appeared to have few phenotypic differences compared to healthy volunteers, yet several individual leukocyte phenotypes within this group correlated with ALS clinical parameters. Conversely, patients clustered in Profile 2, had significant immune abnormalities, both in the overall extent of the number of phenotypes and to the degree in which they were altered. Intriguingly, the dramatically altered immune system in Profile 2 patients was associated with younger age, familial forms of ALS, and significantly prolonged survival.

While it is still unclear whether neuroinflammation causes ALS, neuroinflammation is thought to greatly influence the rate of disease progression through the balance of both protective and harmful immune responses[22]. Although our reported immune alterations in ALS patients likely correspond to either harmful or protective immune responses, additional work is required to fully characterize the role for each immune marker as there are other conceivable explanations for our results. First, it is possible that we identified immune cells that are merely reacting to abnormal immune activation, but do not necessarily have a neuroprotective role. For example, CD14^+^HLA-DR^lo/neg^ monocytes may serve this role as they are quite capable of reducing localized inflammation. Second, the dramatic differences in the immune cell populations between the two ALS immune profiles may primarily reflect genetic differences between the profiles, especially since Profile 2 has increased familial ALS cases. It is interesting to consider that the immune system may play a different role in familial versus sporadic ALS. Other models of neurodegeneration suggest that immune activation, not immune suppression, may be necessary for controlling chronic neuroinflammation[23]. Our data could potentially support this model in that the patients in Profile 2 survived longer, but had much higher peripheral blood levels of immune effector cells.

Although previous studies have demonstrated alterations of circulating immune cell populations in ALS patients, the results have been inconsistent[24]. Regulatory T cells (Tregs) appear to be the predominant leukocyte phenotype of interest in ALS due to its associations with progression and survival[25–27]. However, the methodologies in which Tregs have been measured and/or reported make interpretation of the data less than straight-forward. Henkel et al reported that CD4^+^CD25^high^ Tregs, as a percent of total leukocytes, were not different between ALS and controls but were lower in rapidly progressing patients[25]. Two other studies reported that Tregs measured as a percent of lymphocytes were significantly lower in ALS patients than in controls and also were associated with disease severity[26, 27] and duration[27]. We found that Tregs were not different in terms of percent of CD4+ T cells or cell counts (cells/μl) between ALS patients and HVs. However, Treg cell counts, but not Tregs as a percent of CD4+ T cells, increased as ALSFRS-R score decreased. We measured Tregs in both percentages of parent populations and cell counts to determine whether Tregs are truly changing in cell numbers and not changing due to variations in the parental population (i.e. the denominator). We have previously reported that context matters in flow cytometry and measuring cell populations as only percentages can lead to misleading data[28]. In relation to these other studies, the increase in Tregs may be explained in part because there are expansions of the denominator cells (e.g. granulocytes or lymphocytes) in ALS patients, or alterations of the denominator cells through the course of the disease as we show in this study. To some degree, our data does support the concept that CD4^+^ T cells serve a protective role in ALS[6]. CD4^+^ T cells do not show an age related decline in ALS patients and immunosuppressive phenotypes like Tregs, CTLA-4^+^, and PD-1^+^ CD4^+^ T cells appear to be induced as the disease progresses. In addition, patients in Profile 1 that had a high percentage of PD-1^+^ CD4 T cells survived longer than those with lower PD-1^+^ CD4 T cells. Since the PD-1 signaling pathway has been shown to restrain T cell activation[29], PD-1^+^ CD4 T cells may thus serve in a neuroprotective role. Myeloid cells also appear to be de-regulated in ALS. CD14^+^CD16^-^ monocytes have been shown to exhibit an inflammatory microRNA signature in ALS patients that are specifically recruited to the spinal cord via the CCL2 pathway[30]. In addition, the neutrophil to monocyte (CD16^-^) ratio was found to be increased in ALS patients and also associated with disease progression[31]. Finally, our data may also help explain the variation in human studies that is just due to the nature of the ALS patient population itself. As such, the outcomes of human studies and clinical trials are likely to be influenced by the ratio of patients in these two immune profiles. Moving forward, it is not unreasonable to expect that these patients may respond differently in clinical trials, especially those testing immunotherapeutic approaches.

Our approach has revealed a wide array of immune alterations in ALS patients. Some immune cells associated with clinical parameters whereas many did not. Conversely, many markers were not different between HVs and ALS patients but did associate with clinical parameters in ALS patients. Additional work will be required to assess the role of these markers and whether they drive destructive or protective immunity or how they might merely be responding to abnormal immune activation. The data presented here are from one blood sample. Therefore, we are continuing to confirm these findings with additional patients and further testing how stable the patients’ immunophenotypes are over time in longitudinal studies with multiple time points. Since the bioinformatics based immune profiling exists in the size of the database, increasing the size of the database may reveal other immune profiles that did not yet have sufficient individuals to reach significance.

One of the immune cells that had the highest degree of change in ALS patients was the abundance of CD3^+^CD56^+^ T cells. CD3^+^CD56^+^ T cells have functions of both NK cells and T cells and can fall into a number of different classifications: cytokine-induced killer cells, activated or innate CD8^+^ T cells, and NK-like or NKT-like cells. CD3^+^CD56^+^ T cells are highly cytotoxic and mediate cell killing in both MHC-restricted and TCR-independent mechanisms[32–35]. Gain of CD56 expression on CD8^+^ T cells has been associated with loss of CD28 expression in aging individuals[20]. CD8^+^CD56^+^CD28^-^ T cells may represent functionally active immunosenescent cells and accumulate due to repeated stimulation of CD56^-^ CD28^+^ T cells. From our flow data, we confirmed that CD56^+^ cells had an enriched population of CD28^-^ cells in both CD4 and CD8 compartments. However, the accumulation of CD3^+^CD56^+^ T cells was not correlated with age in either ALS patients or HV patients. The loss of CD28 on CD56^+^ T cells was quite variable and it appeared that CD4 cells were more likely to have a total loss of CD28. However, due to the wide variation of CD28 expression, we did not detect associations of CD28 loss to any clinical characteristics. Despite this fact, the highly cytotoxic nature of CD3^+^CD56^+^ T cells makes them an intriguing target to further investigate the role of these cells causing and/or potentiating tissue destruction in ALS patients. Unlike CD3^+^CD56^+^ T cells, the loss of HLA-DR on monocytes was not different between healthy volunteers and the entire ALS patient cohort. However, the loss of HLA-DR on monocytes appeared to be more pronounced in patients in Profile 2, where high CD14^+^HLA-DR^lo/neg^ monocytes associated with slower progression of disease. Peripheral blood monocytes likely participate in pro-inflammatory pathways early in the disease but then are likely reprogrammed to become immunosuppressive. HLA-DR expression declines as monocytes sense inflammatory signals much like what happens in monocyte-mediated immune suppression in wound healing and cancer. We have previously demonstrated that the loss of HLA-DR is associated with the immunosuppressive activity of monocytes (CD14^+^HLA-DR^lo/neg^) in cancer patients[17, 36]. In ALS patients we observed that the loss of HLA-DR associated with the increase in granulocytes and PD-1^+^ CD4 T cells with the slope of disease progression in the patients that fell in the abnormal immune profile (Profile 2). Interestingly, we have also observed the relationship of CD14^+^HLA-DR^lo/neg^ monocytes with granulocytes in cancer patients [13, 37]. Taken together, the identification of these immune cells in ALS patients provides biomarkers reflecting disease severity and progression, and potentially new mechanistic insights into the role of neuroinflammation in ALS.

To our knowledge, our study is the most comprehensive data set of potential peripheral blood biomarkers in ALS to date. We hope that this data is useful to propose confirmatory studies on the biomarker capacity to diagnose and monitor the disease, suggest new mechanistic studies in the pathology of ALS, and implicate new targets for immune-based therapies tailored to the unique aspects of immune alterations in ALS. This approach could also be used to establish a comprehensive database for patients with neurodegenerative disease to improve the mechanistic, prognostic, and treatment of these multifaceted diseases.

## Supporting information

S1 Fig Associations of phenotypes with principal components. (PDF)

S1 Table Comparison of immunophenotypes between healthy volunteer controls and ALS patients. (PDF)
