# Supplementary material for: Comprehensive immune profiling reveals substantial immune system alterations in a subset of patients with amyotrophic lateral sclerosis
Source: PLoS One. 2017 Jul 25;12(7):e0182002. doi: 10.1371/journal.pone.0182002 (PMC5526569; doi:10.1371/journal.pone.0182002)
Supplement: S1 Fig — (PDF) [file pone.0182002.s001.pdf]

A

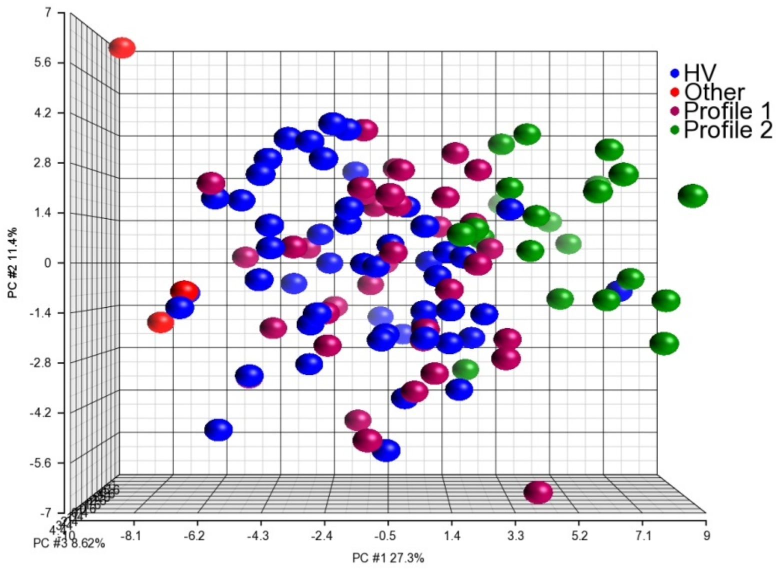

B

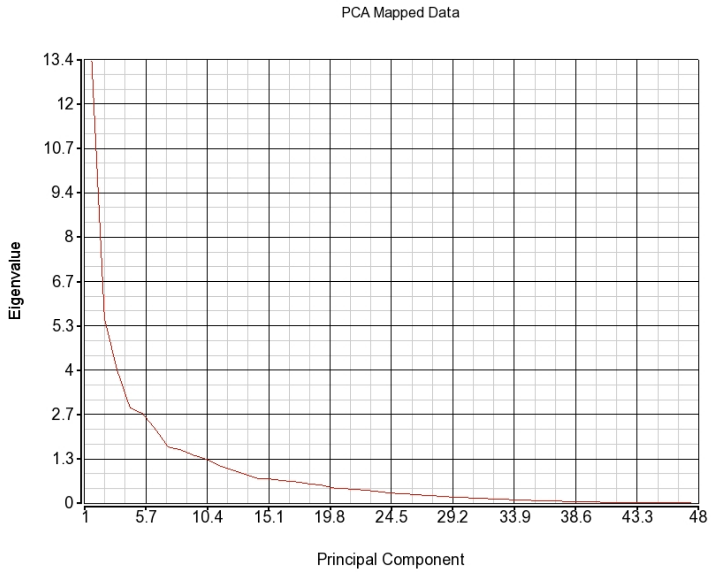

C

PC1		PC2		PC3	
Phenotype	Correlation	Phenotype	Correlation	Phenotype	Correlation
CD3+	0.9221	CD3+CD56+	0.5060	CD14+CD16- HLA-DR	0.7314
CD4+	0.8362	CD8+CD45RA+	0.4983	CD14+CD16+ HLA-DR	0.7056
CD4+CD28+	0.8147	CD8+	0.4846	CD14loCD16+ HLA-DR	0.6900
CD4+PD1-	0.7792	CD8+PD1-	0.4839	CD14loCD16-	0.4773
CD8+CD28+	0.7503	CD8+CD28+	0.4447	CD14+CD16+	0.4351
CD8+	0.7454	CD4+CD8+	0.3364	LINDR+	0.4282
CD19+	0.7285	CD8+PD1+	0.3149	MoDC	0.3839
Tregs	0.7256	CD14+CD16- HLA-DR	0.2593	CD8+PD1+	0.3084
CD19+CD21+	0.7040	gd T cells	0.2366	CD4+PD1+	0.2790
CD8+PD1-	0.6722	CD8Tem	0.2360	CD4Tem	0.2707

S1 Fig. Associations of phenotypes with principal components. A. Principal components scatter plot of HVs and ALS patients by profile. For graphical purposes HVs were colored differently (Blue spheres) than ALS patients. All but two HVS were categorized into Profile 1. ALS patients were colored by profile: Profile 1= purple spheres; Profile 2 = green spheres; and Other (did not cluster into a distinct group) = red spheres. **B.** Eigenvalues that correspond to the variation found in the principal components. The largest eigenvalues are associated with most of the covariation observed in the data set. **C.** For the correlation matrix, the top ten immunophenotypes associated with each principal component are listed.
