# Supplementary material for: Comprehensive immune profiling reveals substantial immune system alterations in a subset of patients with amyotrophic lateral sclerosis
Source: PLoS One. 2017 Jul 25;12(7):e0182002. doi: 10.1371/journal.pone.0182002 (PMC5526569; doi:10.1371/journal.pone.0182002)
Supplement: S1 Table — (PDF) [file pone.0182002.s002.pdf]

S1 Table: Comparison of immunophenotypes between healthy volunteer controls and ALS patients.

	HVs			ALS					
Phenotype	Minimum	Maximum	Mean	Minimum	Maximum	Mean	P value	sig	Discovery?
SShiCD15+ Granulocytes (cells/µl)#	1570	5689	3007	1589	10711	4024	0.000042	*	Yes
CD15+CD16+ Neutrophils (cells/µl)	1487	5557	2802	1440	6481	3556	0.000258	*	Yes
CD15+CD16- Eosinophils (cells/µl)	43.47	1240	198.9	8.15	487	153.5	0.123051		No
Mononuclear cells (cells/µl)	1210	4001	2073	981.4	4768	2667	0.000021	*	Yes
CD14+ Monocytes (cells/µl)#	245.4	838.5	490.8	153	1245	519.8	0.355138		No
CD14+CD16- Classical Monocytes (cells/µl)	184.9	702.6	408.1	157.3	986.6	441.4	0.224147		No
CD14+CD16+ Intermediate Monocytes (cells/µl)	11.56	77.83	35.1	8.86	134.6	33.16	0.607277		No
CD14loCD16+ Non-Classical Monocytes (cells/µl)	20.97	78.67	41.38	12.19	151.8	47.01	0.185053		No
Lymphocytes (cells/µl)	822.5	3403	1577	780.3	4073	2133	0.00001	*	Yes
CD3+ T cells (cells/µl)#	460.2	2461	1090	565.3	3103	1461	0.000086	*	Yes
CD4+ T cells (cells/µl)#	307.7	1883	764.2	360.5	2227	997	0.000309	*	Yes
CD8+ T cells (cells/µl)#	88.7	571.7	294.1	38.63	1308	413.6	0.003012	*	Yes
CD4+CD8+ T cells (cells/µl)	1.48	34.29	9.858	1.25	258	26.71	0.004934	*	Yes
CD4-CD8- T cells (cells/µl)	4.9	56.92	23.2	5.11	194.2	40.03	0.006146	*	Yes
CD4/CD8 ratio#	0.98	10.42	3.085	0.53	14.27	3.292	0.584853		No
CD3+CD56+ NKT cells (cells/µl)#	2.2	170.8	36.09	0.44	579.7	81.11	0.000863	*	Yes
CD3+CD56+ NKT cells (% of CD3+)#	0.34	14.62	3.366	0.07	23.49	5.193	0.01075	*	Yes
gamma delta T cells (cells/µl)	2.49	46.56	15.4	1.6	184.1	33.09	0.003629	*	Yes
gamma delta T cells (% of CD3+)	0.28	4.03	1.392	0.12	7.92	2.092	0.02607	*	Yes
CD19+ B cells (cells/µl)#	63.89	657.8	202.7	66.26	511.1	238.5	0.080178		No
CD56+CD16+ NK cells (cells/µl)#	51.8	436.9	181.9	64.27	898.5	243	0.005503	*	Yes
CD16+CD56- NK cells (cells/µl)	7.06	43.85	21.37	1.54	117.4	23.93	0.39645		No
CD56+CD16+ NK cells (cells/µl)	34.63	375.5	163.9	58.26	882.7	223	0.010625	*	Yes
CD56+CD16- NK cells (cells/µl)	6.9	61.45	17.95	6.01	70.46	20	0.30348		No
Lineage neg cells (cells/µl)	35.48	171	79.83	24.63	218.4	83.43	0.507227		No
CD4+CD25+CD127lo Tregs (% of CD4+ T cells)	4.85	14.74	9.131	4.65	15.98	9.257	0.798762		No
CD4+CD45RA+ (% of CD4+ T cells)	7.57	71.23	42.49	10.22	72.35	40.21	0.391058		No
CD4+CD45RO+ (% of CD4+ T cells)	8.97	91.13	53.09	25	88.24	56.39	0.246014		No
CD4+CD45RO+CD62L+CCR7+ Tcm (% of CD4+CD45RO+ T cells)	28.22	80.02	61.98	37.79	85.81	63.21	0.585035		No
CD4+CD45RO+CD62L-CCR7- Tem (% of CD4+CD45RO+ T cells)	5.4	53.2	17.92	4.3	44.2	16.44	0.451882		No
CD4+CD45RA+CD62L+CD27+	76.82	99.79	94.27	46.1	99.83	92.71	0.383328		No
CD4+CD25+ (% of CD4+ T cells)	12.36	53.19	29.39	2.04	84.82	31.27	0.519229		No
CD4+CD69+ (% of CD4+ T cells)	0.73	46.64	9.369	0.98	30.89	8.744	0.729823		No
CD8+CD45RA+ (% of CD8+ T cells)	23.81	81.79	56.2	22.3	90.25	53.55	0.356893		No
CD8+CD45RO+ (% of CD8+ T cells)	1.59	75.41	41.02	8.95	77.3	44.86	0.20659		No
CD8+CD45RO+CD62L+CCR7+ Tcm (% of CD8+CD45RO+ T cells)	4.38	47.73	23.12	6.24	42.59	22.37	0.665672		No
CD8+CD45RO+CD62L-CCR7- Tem (% of CD8+CD45RO+ T cells)	28.29	88.43	53.77	29.83	78.71	52.54	0.606539		No

CD8+CD45RA+CD62L+CD27+ (% of CD8+CD45RA+)	9.34	89.09	52.21	8.79	94.56	43.98	0.067497		No
CD8+CD45RA+CD62L-CD27- Temra (% of CD8+CD45RA+)	0.23	45.1	14.36	0.43	61.86	17.33	0.291855		No
CD8+CD25+ (% of CD8+ T cells)	0.62	14.53	4.88	0.57	65.61	10.44	0.003601	*	Yes
CD8+CD69+ (% of CD8+ T cells)	1.43	24	6.243	1.44	21.59	5.479	0.323901		No
CD8+CD45RA+CD62L+CD27+CCR7+ Tscm (% of CD8+CD45RA+CD27+)	2.93	66.66	24.33	2.8	54.19	17.54	0.01272	*	Yes
CD4+CTLA4+ (% of CD4+ T cells)	0.01	3.42	0.4308	0	3.15	0.3572	0.567599		No
CD4+CD28+CTLA4+ (% of CD4+ T cells)	0.06	12.26	1.586	0	15.78	2.663	0.049923	*	No
CD4+CD28+ (% of CD4+ T cells)	59.75	99.21	91.44	71.92	99.37	92.01	0.700046		No
CD8+CTLA4+ (% of CD4+ T cells)	0	10.29	0.3835	0	2.5	0.2272	0.455088		No
CD8+CD28+CTLA4+ (% of CD8+ T cells)	0	10.49	1.096	0	7.71	1.269	0.660268		No
CD8+CD28+ (% of CD8+ T cells)	47.45	99.08	87.11	58.57	99.57	86.49	0.812437		No
CD4+TIM-3+ (% of CD4+ T cells)	0.2	10.12	1.563	0.2	17.04	2.836	0.064667		No
CD4+PD-1+TIM-3+ (% of CD4+ T cells)	0.12	6.52	1.036	0.1	9.97	1.627	0.10767		No
CD4+PD-1+(% of CD4+ T cells)	6.92	40.66	20.47	5.86	40.88	20.05	0.797587		No
CD8+TIM-3+ (% of CD8+ T cells)	0.02	3.07	0.54	0.02	6.57	1.101	0.020879	*	Yes
CD8+PD-1+TIM-3+ (% of CD8+ T cells)	0.08	13.15	1.285	0.02	15.52	2.713	0.020197	*	Yes
CD8+PD-1+ (% of CD8+ T cells)	18.93	71.88	37.58	8.25	75.57	37.16	0.879212		No
CD4+BTLA+ (% of CD4+ T cells)	56.75	92.36	81.45	58.63	93.84	80.41	0.46179		No
CD4+BTLA PE Geometric Mean	0.61	4.01	2.193	0.5	4.7	2.178	0.931017		No
CD8+BTLA+ (% of CD8+ T cells)	32.77	91.48	66.68	35.18	85.83	62.8	0.171564		No
CD8+ BTLA PE Geometric Mean	0.29	4.74	1.676	0.48	4.54	1.463	0.251403		No
CD19+CD21- Gated (% of CD19+ B cells)	2.82	40.58	9.074	1.75	48.96	9.395	0.816204		No
CD19+CD21pos+ (% of CD19+ B cells)	59.42	97.18	90.93	51.04	98.25	90.61	0.816204		No
CD19+CD27+ (% of CD19+ B cells)	9.26	54.26	26.82	9.13	80.79	28.46	0.513475		No
CD19+IgM+ % Gated (% of CD19+ B cells)	22.08	92.05	72.09	15.61	94.35	59.54	0.000811	*	Yes
CD19+CD24+IgM- Plasma (% of CD19+ B cells)	7.91	61.61	26.03	4.77	76.94	35.23	0.004116	*	Yes
CD19+CD24+IgM+ Transitional (% of CD19+ B cells)	20.48	91.49	69.5	18.04	93.85	61.35	0.023329	*	Yes
CD19+IgD+IgM+ (% of CD19+CD27+)	3.46	35.04	11.16	3.37	47.37	11.43	0.845768		No
CD19+IgD-IgM+ (% of CD19+CD27+)	0.37	15.63	4.789	0.41	36.44	5.543	0.447415		No
CD19+IgD-IgM- (% of CD19+CD27+)	3.08	26.29	10.2	1.38	31.97	10.21	0.99111		No
CD14+CD86+ (% of CD14+)	88.75	99.49	96.72	77.19	99.72	95.66	0.098496		No
CD14+CD80+ (% of CD14+)	0.13	9.91	1.661	0.11	13.32	1.463	0.618809		No
CD14+CD16- Classical (% of CD14+)	62.98	92.52	79.97	69.95	93.93	81.54	0.117203		No
Classical HLA-DR PacBlu Geometric Mean	0.84	6.35	3.552	0.98	7.41	3.307	0.35191		No
CD14+CD16+ Intermediate (% of CD14+)	1.96	29.37	7.955	1.49	16.68	7.156	0.284271		No
Intermediate HLA-DR PacBlu Geometric Mean	3.07	37.25	19.15	1.87	35.19	15.09	0.0155	*	Yes
CD14dimCD16+ Non-classical (% of CD14+)	2.01	17.48	7.421	1.61	16.44	6.32	0.090371		No
Non-Classical HLA-DR PacBlu Geometric Mean	3.14	18.57	8.593	1.55	18.82	8.558	0.960308		No
CD14+CD142+ (% of CD14+)	0.07	6.54	1.02	0.09	10.79	1.177	0.58129		No

CD14+HLA-DRlo/neg (% of CD14+)	2.2	62.31	15.26	1.43	55.07	16.91	0.50686		No
CD14+ HLA-DR PacBlu Geometric Mean	0.97	7.52	4.099	1.17	6.82	3.7	0.187278		No
Classical CD86 APC Geometric Mean	3.05	12.44	7.04	2.09	13.67	6.143	0.068108		No
Intermediate CD86 APC Geometric Mean	0.88	33.5	14.45	0.72	32.68	12.07	0.079479		No
Non-Classical CD86 APC Geometric Mean	0.11	44.04	14.44	0.11	44.71	12.62	0.305381		No
Classical CD80 FITC Geometric Mean	0.48	1.29	0.791	0.36	1.29	0.6386	0.00147	*	Yes
Intermediate CD80 FITC Geometric Mean	0.53	1.5	0.9625	0.41	1.59	0.7924	0.003817	*	Yes
Non-Classical CD80 FITC Geometric Mean	0.41	2.74	0.9588	0.3	6.15	0.8392	0.391558		No
CD14+ CD32 PerCP-e710 Geometric Mean	0.11	7.44	4.57	1.06	8.15	4.324	0.464909		No
Neutrophils (% of CD15+)	54.22	98.09	86.38	48.83	98.62	86.11	0.892084		No
Eosinophils (% of CD15+)	1.69	38.02	11.94	1.16	48	11.44	0.769954		No
CD203c+ Basophils (% of CD45+)	0.03	0.59	0.1647	0.01	6.18	0.2416	0.509143		No
CD203c+CCR3+CD63+ (% of CD203c+)	8.45	91.13	40.54	3.65	90.2	36.01	0.261777		No
CD203c+CCR3-CD63- (% of CD203c+)	6.28	90.61	55.51	9.8	95.96	59.82	0.282064		No
CCR3+CD49d+ Eosinophils (% of Eosinophils)	34.68	97.92	83.23	9.46	96.28	74.58	0.008135	*	Yes
CCR3-CD49d- Eosinophils (% of Eosinophils)	0.65	46.96	8.314	1.14	48.77	13.04	0.018968	*	Yes
CCR3+CD49d+ Eosinophils CD44 PerCp PC5.5 Geometric Mean	5.53	21.85	11.35	2.01	17.65	11.12	0.716807		No
CCR3-CD49d+ Eosinophils CD44 PerCp PC5.5 Geometric Mean	0.5	3.79	1.094	0.25	4.16	1.318	0.158716		No
Neutrophils CD66b FITC Geometric Mean	6.85	18.56	12.16	5.64	19.38	10.22	0.000861	*	Yes
CCR3+CD49d+ Eosinophils CD66b FITC Geometric Mean	13.14	32.8	21.77	10.22	33.31	20.19	0.163387		No
CCR3-CD49d- Eosinophils] CD66b FITC Geometric Mean	0.89	8.89	1.834	0.51	6.15	1.65	0.388147		No
CD15+ G-MDSC _(% of Mononuclear cells)	0.09	4.03	0.6304	0.06	2.91	0.564	0.587069		No
G-MDSC CD66b FITC Geometric Mean	0.23	6.75	1.588	0.16	7.95	1.582	0.983861		No
LIN-CD33+HLA-DR- (CD11b++CD16-CD15-CD66b-) Immature MDSCs (% of Mononuclear cells)	0.16	5.63	2.526	0.05	5.01	2.019	0.009094	*	Yes
Lineage(-)DR+ (% of Mononuclear cells)	1.31	5.87	3.262	0.57	4.79	2.463	0.000066	*	Yes
LIN-DR+CD33+CD11c++CD16+ (% of Lineage(-)DR+)	9.87	77.23	50.24	0.28	82.46	39.92	0.008679	*	Yes
LIN-DR+CD33+CD11c++CD16- (% of Lineage(-)DR+)	7.91	77.3	28.8	7.65	90.18	38.51	0.006025	*	Yes
LIN-DR+CD11c dim (% of Lineage(-)DR+)	1.58	12.26	3.994	0.47	32.48	4.873	0.256504		No
CD11cdimCD123br (% of Lineage(-)DR+)	4.05	31.03	15.51	1.95	46.72	14.73	0.614179		No
CD15+SSClo (% of Mononuclear cells)	0.2	25.81	4	0.07	43.43	4.916	0.513796		No
CD15+SSCloCD66b+ (% of CD15+)	0.03	6.61	1.061	0	6.17	1.04	0.926508		No
Classical TNFR2 PE Geometric Mean	0.13	13.65	4.201	0.33	12.5	3.798	0.493665		No
Intermediate TNFR2 PE Geometric Mean	0.41	18.96	8.326	1.16	16.52	7.413	0.302125		No
Non-Classical TNFR2 PE Geometric Mean	1.87	21.05	8.74	1.87	23.1	8.515	0.774692		No
Classical CD40 APC Geometric Mean	0.53	1.41	0.8492	0.44	2.34	0.8188	0.49303		No
Intermediate CD40 APC Geometric Mean	0.82	9.89	2.52	0.26	6.83	2.097	0.150457		No
Non-Classical CD40 APC Geometric Mean	0.11	8.01	2.699	0.11	7.39	3.326	0.144861		No

Includes 17 patients performed with 4-color flow protocols
